# Measurement Properties of the Full and Brief Version of the Work Rehabilitation Questionnaire in Persons with Physical Disabilities

**DOI:** 10.1007/s10926-021-09973-8

**Published:** 2021-04-12

**Authors:** Ellen H. Roels, Michiel F. Reneman, Marcel W. M. Post

**Affiliations:** 1 Department of Rehabilitation Medicine, Center for Rehabilitation, University of Groningen, University Medical Center Groningen, Groningen, The Netherlands; 2 Center of Excellence for Rehabilitation Medicine, UMC Utrecht Brain Center, University Medical Center Utrecht and De Hoogstraat Rehabilitation, Utrecht, The Netherlands

**Keywords:** Questionnaire, Vocational rehabilitation, Employment, Spinal injury, Stroke

## Abstract

*Purpose* The Work Rehabilitation Questionnaire (WORQ) is a self-report vocational rehabilitation assessment. A comprehensive (WORQ-FULL) and a brief version (WORQ-BRIEF) are available. The purpose of this study was to investigate measurement properties of both versions in persons with physical disabilities. *Methods* Cross sectional and test–retest design. Adults with physical disabilities in vocational rehabilitation were included. Internal consistency (Cronbach’s alpha), test–retest reliability (intra-class correlation; ICC), agreement between sessions (Bland–Altman Plots), criterion validity (ICC and agreement with Bland–Altman Plots between WORQ-FULL and WORQ-BRIEF) and convergent validity with the Work Ability Index -Single item (WAS) and the EuroQOL 5D-5L were analyzed. *Results* Out of the 91 individuals who agreed to participate, 74 (81%) returned questionnaire T1 and 49 (54%) participants returned questionnaire T2 within the maximum time interval (= 27 days). At T2, 28 (57%) participants reported no medical changes compared to T1. Median age was 49 (IQR 40–60), 57% were male, 47% had experienced a stroke and 27% a spinal cord injury (n = 49). Internal consistency was good: 0.95/0.95/0.94 for the WORQ-FULL and 0.88/0.89/0.85 for the WORQ-BRIEF (n = 74/n = 48/n = 28, respectively). Test–retest reliabilitywas good: ICC = 0.86/0.85 for the WORQ-FULL and ICC = 0.87/0.86 for the WORQ-BRIEF (n = 49/ n = 28). Bland Altman plots suggested a higher score at T1. As for criterion validity of the WORQ-FULL versus the WORQ-BRIEF, ICC was good (ICC = 0.84; n = 74), however Bland Altman plots indicated potential bias. Correlations with the WAS/EuroQOL 5D-5L were variable: r = -0.24/r = -0.57 (WORQ-FULL) and r = -0.28/-0.65 (WORQ-BRIEF). *Conclusions* The WORQ showed good internal consistency and test–retest reliability. Agreement demonstrated large score differences are needed to indicate change beyond random chance at individual level, whereas small changes are sufficient at group level. Criterion validity of the WORQ-FULL versus the WORQ-BRIEF was supported, however, agreement demonstrated moderate to large score differences are needed to indicate change beyond random chance at individual level, whereas small changes are sufficient at group level. This indicates the WORQ-FULL and WORQ-BRIEF are better not used interchangeably. Correlation analyses provided better insight in the validity of the WORQ. Convergent validity was supported for the WORQ-BRIEF with the EuroQoL 5D-5L (r = -0.65).

## Introduction

Work participation (WP) of persons with a physical disability has many advantages for the individual and society [1–4]. Employment rates of persons with disabilities, however, remain low compared to the general population with reported averages of 35% among people with spinal cord injury (SCI) [5] and 67% among people with stroke [6]. Vocational rehabilitation (VR) can be instrumental to enhance WP outcomes after onset of physical disability. A biopsychosocial approach to VR is warranted to address all aspects of the return to work (RTW) process [7].

A VR Core Set including a selection of categories from the International Classification of Functioning, Disability and Health (ICF) [8] that were considered the most relevant to describe functioning of VR clients was developed for use in VR practices [9]. Based on this VR Core Set, the Work Rehabilitation Questionnaire (WORQ) was developed to assess work-related functioning [10]. The WORQ can be used to describe the client’s level of functioning on work related domains at the start of VR; to set VR goals and monitor functioning over time; to facilitate interdisciplinary communication in VR; to stimulate the patient to actively participate in his/her VR process; and to open up a conversation with the rehabilitant simplifying communication [11]. Moreover, the WORQ questionnaire is free and easy to use. The first version of the WORQ was interview administered. The initial psychometric evaluation of the WORQ showed satisfactory test–retest reliability and good internal consistency [10]. To improve its practicality, a self-report version was developed [12]. This version suggested content validity in a sample of people with SCI in the early subacute phase [12]. This version has been translated in many languages [13], including French and Dutch (Flemish version for use in Belgium) [14, 15]. Both translations showed good internal consistency (Cronbach’s alpha 0.97 and 0.95 respectively) and good test–retest reliability (ICC 0.94 and 0.85 respectively) in relatively small samples with varying pathologies. Studies into the construct validity of the WORQ have revealed correlations with several reference measures ranging from 0.28 to 0.81 [10, 14–17]. Based on the full version of WORQ, a brief 13-item version was developed to encounter the challenge of the length of the WORQ [14]. A shorter version may be preferred if it has adequate measurement properties and correlates strong with the comprehensive version. The brief version has shown good internal consistency (Cronbach’s alpha 0.96) and test–retest reliability (ICC 0.91) [17], but agreement between the full and the brief version of the WORQ has not yet been investigated.

Because research in different diagnostic groups is needed to assess the validity of the WORQ [17], this study was conducted to test the internal consistency, test–retest reliability, agreement, and convergent validity of the full and brief versions of the WORQ for use in the Netherlands (WORQ-NL) in persons with physical disabilities in a VR setting.

## Methods

### Design

A cross-sectional and test–retest design was applied. Participants first completed questionnaire T1 (= WORQ-NL part 1 and 2, Work Ability Index -Single item (WAS) and EuroQol-5D-5L) and 1 week after receiving questionnaire T1 by mail, questionnaire T2 (= control questions, WORQ-NL part 2 and WAS) was sent. Inclusion of 50 persons was aimed at, based on proposed quality criteria for this type of reliability studies [18]. Data collection took place between April 2018 and July 2019.

### Participants

This study included persons, aged 18 to 65 years old, with physical disabilities (spinal cord injury, brain injury, stroke, neuromuscular disease, multiple sclerosis, amputation, or other) who had been referred for vocational rehabilitation (VR) at the Centre for Rehabilitation, University Medical Center Groningen, location Haren, The Netherlands. The participants had to be able to read and write the Dutch language.

### Procedures

The study was introduced by the vocational counselor who gave oral and written information during the intake consultation. Once informed consent was given, the participant received the questionnaire, an instruction leaflet and a return envelope. The participant was requested to fill out the questionnaire independently and return it by postal mail to the researcher. Approximately 1 week after arrival of questionnaire T1, questionnaire T2 with a return envelope was sent to the participant by postal mail, with the request to fill out the questionnaire independently and return it to the researcher within 1 week. No reminder was sent. If questionnaire T2 was filled out 28 or more days after questionnaire T1 was filled out, the participant was excluded because too long time interval between both questionnaires. To trace possible changes in health and work status during the time interval, the following control questions were asked in the second questionnaire: ‘’Did anything regarding your medical condition change during the past 2 weeks when compared to the weeks before? If yes, please specify.’’ and ‘’Did anything regarding your work situation change during the past 2 weeks when compared to the weeks before? If yes, please specify.’’.

### Instruments

#### WORQ

The two versions of the WORQ were used: the 40 items questionnaire (further mentioned as WORQ-FULL) and the brief version questionnaire (further mentioned as WORQ-BRIEF). The WORQ consists of two parts. Part 1 is identical for the WORQ-FULL and WORQ-BRIEF, and has 17 questions collecting socio-demographic and work-related information. Part 2 of the WORQ-FULL consists of 40 evaluative questions (18 body functions and 22 activities and participation items) whereas part 2 of the WORQ-BRIEF consists of 13 evaluative questions (6 body functions and 7 activities and participation items) [14, 17]. The questions in part 2 are answered on a numerical rating scale ranging from 0 (no problem) to 10 (complete problem).

The WORQ-NL was developed for use in The Netherlands in collaboration with the developers of the Flemish version [15]. Part 1 was substantially adapted since this part contains questions that are country-specific (e.g. education level grading). For part 2, minimal changes in wording of the Flemish version were negotiated and consensus was reached on a single version for use in Flanders and the Netherlands. However, in the WORQ-NL a response option ‘not applicable’ was added to question 34 (driving) for people without driving license, without a car. The sum score of the WORQ-FULL was calculated as the sum of the 40 questions of part 2 (higher scores indicate higher work-disability). The sum score of the WORQ-BRIEF was calculated from the WORQ-FULL by adding the scores of the related 13 questions. Furthermore the mean item score (sum score/ n of items) was computed.

#### WAS

The Work Ability Index (WAI) is a well-known instrument used in occupational health care and research to assess work ability of workers during health examinations and workplace surveys developed by the Finnish Institute of Occupational Health [19]. The WAS (Work Ability Index – Single item) is derived from the WAI and consists of one question on the participant’s self-reported current work ability compared to his highest work ability ever (scoring from 0 to 10) [20, 21]. The WAS showed a strong association with the WAI and both have shown a strong predictive value for sick leave and health related quality of life [20].

#### Euro-Qol 5D-5L

The Euro-Qol 5D is a simple instrument used to describe health related quality of life. It consists of a self-reported health state description with 5 items (mobility, self-care, usual activities, pain/discomfort, and anxiety/depression) [22]. The Euro-Qol 5D has shown validity and reliability e.g. in the stroke population [22]. The number of answer categories has been increased from 3 levels (EQ-5D-3L) to 5 levels (EQ-5D-5L): 1 = no; 2 = slight; 3 = moderate; 4 = severe problems or 5 = extreme problems [23, 24]; and which has improved its measurement properties [25].

The index valuation score of the EQ-5D-5L was used (a maximal score of 1 stands for perfect health) [26].

### Data from Medical Files

The diagnostic group was the only data from the medical file that was provided to the researcher by the vocational counselor.

### Statistical Analysis

Data were analyzed using SPSS for Windows, version 23.0 (IBM Corp., Armonk, NY). Descriptive analysis were used to describe the study sample. Analyses were performed on the sum-WORQ-FULL—and on the sum-WORQ- BRIEF scores. If a participant provided 2 scores per question, the average of the two scores was used. In case of the EuroQoL, this average was rounded up- or downwards into the nearest integer value. If item 34 was marked as not applicable, this was treated as a missing value. If less than 25% of the item scores were missing, the missing values were replaced by the mean of the valid WORQ item scores. Descriptive, reliability and agreement analyses were repeated in the subgroup of participants not reporting relevant changes in their medical condition. Only few participants mentioned job changes, and these were considered irrelevant for the purpose of this study. Therefore the analyses were not repeated in the subgroup without job changes.

#### Reliability

Internal consistency of the WORQ was examined using Cronbach’s alpha. Values of alpha can range from 0 (no internal consistency) to 1 (perfect internal consistency). Internal consistency was considered good when alpha coefficients are between 0.70 and 0.95 [18].

To analyze test–retest reliability, we used the intraclass correlation coefficient (ICC). The ICC was calculated for the WORQ-FULL T1 versus WORQ-FULL T2 and the WORQ-BRIEF T1 versus WORQ-BRIEF T2.

A two-way mixed effects model for absolute agreement was used [27]. An ICC of > 0.70 indicates sufficient reliability and > 0.80 is good [28].

#### Agreement

A paired samples t-test was performed to test for differences in the sum scores for WORQ-FULL T1 versus WORQ-FULL T2 and for WORQ-BRIEF T1 versus WORQ-BRIEF T2.

The values of the paired samples t-test were used for constructing the Bland–Altman plots. Bland–Altman plots were used to analyze agreement between the sum-WORQ scores at T1 and T2. The limits of agreement (LOA) were computed, defined as ± 1.96SD of the mean difference score. To express the figures in terms of effect size (ES), the LOA was divided by the SD of the baseline score. Similarly the ES at group level was calculated as 1.96 times the standard error of the difference score (SE = SD/√N) divided by the SD of the baseline score [29]. Cohen’s approach was used to interpret these values: 0.2 is small, 0.5 is moderate and 0.8 is large [28].

#### Criterion validity

To test for criterion validity of the WORQ-FULL versus the WORQ-BRIEF, we used the intraclass correlation coefficient (ICC). This was calculated with the mean item score of both versions (sum score/ n of items) as the sum score of the WORQ-FULL (40 items) is obviously considerably higher than the sum score of the WORQ-BRIEF (13 items).

A paired samples t-test was performed to test for differences in means for WORQ-FULL T1 versus WORQ-BRIEF T1. The values of the paired samples t-test were used for constructing Bland–Altman plots. Bland–Altman plots were used to analyze agreement between the WORQ-FULL and BRIEF scores at T1. The LOA and ES was calculated as described above.

#### Validity

Convergent validity was tested with correlational analyses between WORQ scores at baseline with the WAS and EuroQoL scores using the Pearson correlation coefficient. Higher scores on the WORQ were expected to correlate with a lower score on the WAS and with a lower score on the EuroQol (negative correlation). A correlation of > 0.6 was expected between the EuroQoL and WORQ and the WAS and WORQ to support convergent validity [30].

## Results

### Data Collection Process

Out of the 91 individuals who agreed to participate, 74 (81%) and 60 (66%) returned questionnaire T1 and T2 respectively of whom 49 (54%) participants returned questionnaire T2 within the maximum time interval. Out of the 49 participants that filled in questionnaire T2 within the maximum time interval, 57% (28 out of 49) reported medical changes and 6% (3 out of 49) reported job status changes. The reported job changes were considered minor and not potentially disturbing the reliability results (starting 1–2 h of work per week, having a work visit, starting volunteering). The reported medical changes included: less medical issues; chest infection; knee pain; improvement of muscle strength, sensibility, walking, writing or speaking; physical improvement; less or more pain; more energy; improvement of condition; perspective change; better tolerance for stimuli; more emotional; infection and improvement of functioning.

### Descriptive Analysis

The demographic, employment and VR-related characteristics of the participants are displayed in Table 1.

**Table 1:** Participants characteristics

Median (IQR) or n (%)	Total group at baseline n = 74	Test–retest n = 49	Test–retest unchanged n = 28
Age (years)	48 (37.8–56.2)	49 (40–60)	49 (40–60)
Sex (male)	42 (57%)	28 (57%)	16 (57%)
Diagnosis Stroke	33 (45%)	23 (47%)	12 (43%)
Spinal cord injury	17 (23%)	13 (27%)	8 (29%)
Neuromuscular disease	13 (18%)	7 (14%)	5 (18%)
Other	11 (15%)	6 (12%)	3 (11%)
Civil status Married/partnership	49 (66%)	35 (71%)	22 (79%)
Single/divorced/separated/widowed	25 (34%)	14 (27%)	6 (21%)
Work status Employed	56 (76%)	38 (78%)	21 (75%)
Self-employed	6 (8%)	5 (10%)	2 (7%)
Not (formally) employed	12 (16%)	6 (12%)	5 (18%)
Current job status Working full time	5 (7%)	4 (8%)	3 (11%)
Part time	3 (4%)	1 (2%)	0 (0%)
Therapeutic work	8 (11%)	5 (10%)	4 (14%)
*Missing*	*4 (5%)*	*2 (4%)*	*1 (4%)*
Time off work (days)	108 (71–252)	112.5 (64–261)	140 (83–414)
*Missing/NA*	*8(11%)*	*5 (10%)*	*4 (14%)*
Education Primary school	1 (1%)	1 (2%)	1 (4%)
Secondary school	41 (55%)	25 (51%)	14 (50%)
High school	21 (28%)	14 (29%)	10 (36%)
University	9 (12%)	7 (14%)	3 (11%)
*Missing*	*2 (3%)*	*2 (4%)*	*0 (0%)*
In medical treatment (% yes)	66 (89%)	45 (92%)	26 (93%)
*Missing*	*4 (5%)*	*2 (4%)*	*1 (4%)*
Time between questionnaires (days)	NA	14 (12–16)	13.5 (12–15)

*IQR* interquartile range, *n* number, *NA* not applicable

The participants were mainly residing in the community, however also patients undergoing inpatient (n = 2) rehabilitation were included. Median age was 49 (IQR 40–60), 57% were male, 47% had experienced a stroke and 27% spinal cord injury (n = 49). Few missing values were seen, 53 (0.002%) of all answers in WORQ T1 and 12 (0.0006%) of all answers in WORQ T2. Further, 1 participant ticked two boxes in response to one question, once in the WORQ T1 and once in the WORQ T2. Fifteen (20%) participants scored item 34 as not applicable in WORQ T1 and 11 (18%) in WORQ T2.

### Reliability and Agreement

Table 2 shows the mean and the internal consistency values of the WORQ-FULL and the WORQ-BRIEF questionnaires at T1 and T2. Internal consistency was good for both versions and all samples (≥ 0.85). The median score for all measurements was similar to the mean score and is therefore not further mentioned. The mean score of both the WORQ-FULL and the WORQ-BRIEF were lower at T2 when compared with T1. The mean total score (calculated as the mean of the mean item scores) on the WORQ-FULL was lower compared to the corresponding mean total score on the WORQ-BRIEF at both T1 and T2.

**Table 2:** Mean and internal consistency analysis

	WORQ FULL T1	WORQ BRIEF T1	WORQ FULL T2	WORQ BRIEF T2
All n = 74				
Mean (SD)	149.6 (71)*	60.2 (26.5)*		
Mean total score (SD)	3.7* (1.8)	4.6* (2)		
Cronbach's alpha	0.95*	0.88*		
Range sum score	5–345*	3–120*		
Returned n = 49				
Mean (SD)	152.3 (71.3)*	61.1 (27.4)*	132.4 (66.3)	53.1 (25.5)
Mean total score (SD)	3.81* (1.8)	4.7* (2.1)	3.3 (1.7)	4.1 (1)
Cronbach's alpha	0.95*	0.89*	0.95	0.88
No changes n = 28				
Mean (SD)	152.6 (67.8)	61.6 (24.6)	135.4 (61)	54.8 (23.8)
Mean total score (SD)	3.8 (1.7)	4.7 (1.9)	3.4 (1.5)	4.2 (1.8)
Cronbach’s alpha	0.94	0.85	0.93	0.85

*SD* standard deviation

*1 missing

**Fig. 1:**
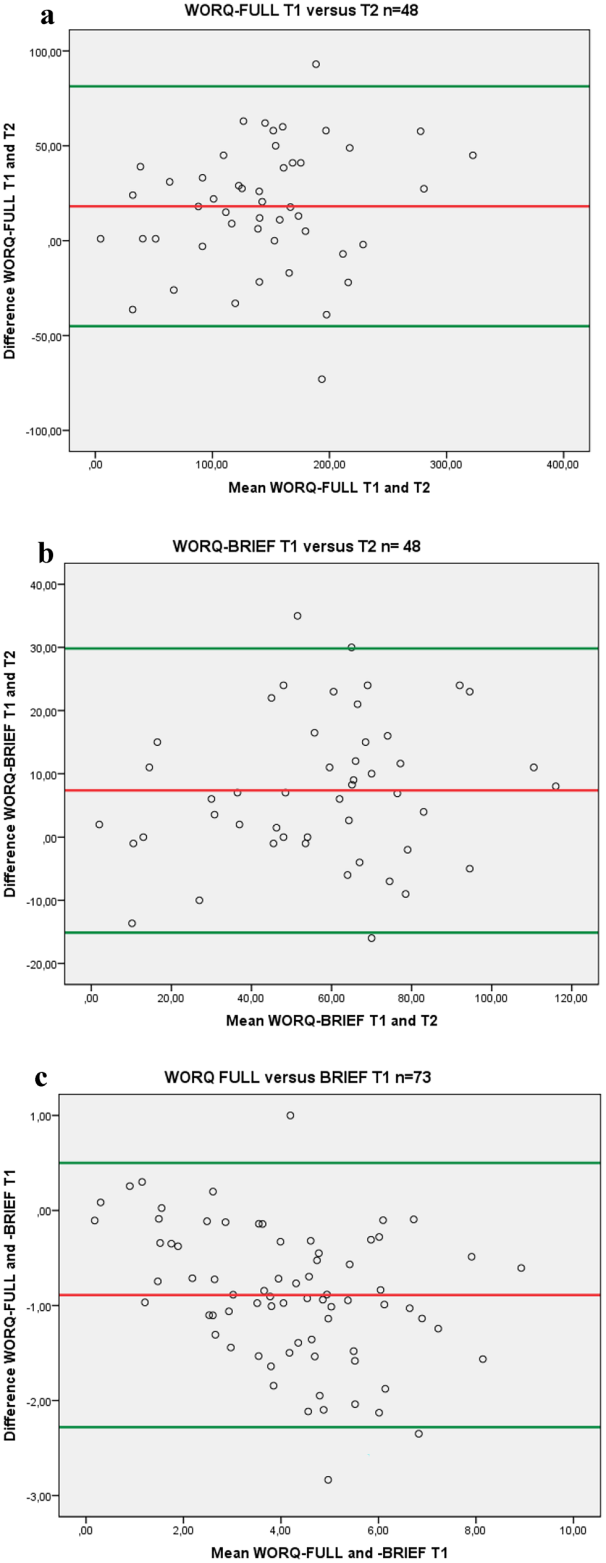
**a–c** Bland–Altman plots of WORQ-FULL T1 vs T2, WORQ-BRIEF T1 vs T2 and WORQ-FULL vs BRIEF T1

Table 3 shows the ICC, paired sample t-test and the LOA values. All ICC values of the WORQ-FULL and the WORQ-BRIEF T1 versus T2 were good (≥ 0.85). ICC for the WORQ-FULL versus WORQ-BRIEF was 0.84 (n = 74). The paired sample t-test showed a statistically significant difference for the WORQ-FULL and the WORQ-BRIEF T1 versus T2 (higher score at T1; p ≤ 0.01) and a statistically significant difference for the WORQ-FULL versus the WORQ-BRIEF (higher score for the WORQ-BRIEF; p = 0.00). Bland–Altman plots are seen in Fig. 1. Because all Bland–Altman plots were similar, only the most relevant are presented, the other plots are available upon request. Participants scored higher at baseline compared to retest, and their mean scores on the WORQ-BRIEF were higher compared to the WORQ-FULL. Visual inspection of these plots suggested no proportional bias for the WORQ-FULL and the WORQ-BRIEF at both time points. However the decreasing trend in Fig. 1c comparing the WORQ-FULL with the WORQ-BRIEF indicated that the higher the scores on the questionnaires, the stronger the difference between the scores on both questionnaires (BRIEF having higher mean score). The LOA were similar and large for all agreement comparisons. For all T1 versus T2 measurements at individual level, large score differences (ES ≥ 0.82) are needed to exceed the limits of agreement indicating change beyond random chance, however at group level small score differences (ES ≥ 0.12) are sufficient to show change beyond random chance. For the WORQ-FULL versus the WORQ-BRIEF at individual level moderate to large score differences (ES = 0.79) are needed to exceed the limits of agreement indicating change beyond random chance, however at group level small score differences (ES = 0.09) are sufficient to show change beyond random change.

**Table 3:** Test–retest reliability, t-test and agreement values of T1 versus T2 and of WORQ-FULL versus WORQ-BRIEF

		WORQ FULL T1 vs T2	WORQ BRIEF T1 vs T2	WORQ FULL T1 vs BRIEF T1**
All n = 74*	ICC (95% CI)			0.84 (0.13–0.95)
	Range of differences	–	–	− 2.83 to 1.0
	Mean of differences (SD)	–	–	− 0.9 (0.7)
	95% CI of difference T1-T2 (p value)	–	–	− 1.05 to − 0.72 (0.00)
	Limits of agreement	–	–	0.50 to − 2.28
	Effect size needed to exceed change I	–	–	0.79
	Effect size needed to exceed change G	–	–	0.09
Returned n = 49*	ICC (95% CI)	0.86 (0.69–0.93)	0.87 (0.67–0.94)	
	Range of differences	− 73 to 93	− 16 to 35	
	Mean of differences (SD)	18.1 (32.3)	7.4 (11.5)	
	95% CI of difference T1-T2 (p value)	8.77–27.50 (0.00)	4.03–10.69 (0.00)	
	Limits of agreement	− 45.08 to 81.34	− 15.12 to 29.84	
	Effect size needed to exceed change I	0.90	0.82	
	Effect size needed to exceed change G	0.13	0.12	
No changes n = 28	ICC (95% CI)	0.85 (0.64–0.93)	0.86 (0.64–0.94)	
	Range of differences	− 39 to 93	− 16 to 30	
	Mean of differences (SD)	17.2 (32.8)	6.8 (11.5)	
	95% CI of difference T1-T2 (p value)	4.509–29.90 (0.01)	2.36–11.24 (0.004)	
	Limits of agreement	− 46.99 to 81.39	− 15.64 to 29.24	
	Effect size needed to exceed change I	0.95	0.91	
	Effect size needed to exceed change G	0.18	0.17	
SCI n = 13	ICC (95% CI)	0.77 (− 0.02 to 0.94)	0.82 (0.14–0.95)	
Stroke n = 23*	ICC (95% CI)	0.88 (0.68–0.95)	0.87 (0.68–0.95)	

*CI* confidence interval, *G* group level, *I* individual level, *ICC* intraclass correlation coefficient, *SD* standard deviation

*1 missing

**Calculated with the mean item score

### Convergent Validity

Correlations of the WORQ-FULL and the WORQ-BRIEF with the EuroQoL 5D-5L were r -0.57 and r = -0.65 respectively (both p < 0.01) and correlation of the WORQ-FULL and the WORQ-BRIEF with the WAS were r = -0.24 and r = -0.28 respectively (both p < 0.05). Convergent validity was supported only for the WORQ-BRIEF with the EuroQoL 5D-5L as we determined a correlation of > 0.6 as cut off value.

## Discussion

This study is the first psychometric testing of the Dutch version of the WORQ (for use in the Netherlands) and also the first to compare the full and the brief versions of the WORQ. The internal consistency was good for both versions in our study. This is in line with results on the WORQ-German (α = 0.88) [10], the WORQ-French (α = 0.97) (14] and the WORQ-VL (α = 0.95) [15]. The results of our study furthermore demonstrate good test–retest reliability for both versions. The ICC values in this study were similar to those observed in previous studies with other language versions or patient samples (0.94 for the WORQ-French [14] and 0.85 for the WORQ-VL [15]). The paired sample t-test however showed a statistical significant difference (higher score at T1; p ≤ 0.01) between the scores for both questionnaires (full and short version). In the Bland–Altman plots this lower sum score in the second measurement is seen for both versions. We could not find previous studies analyzing agreement with Bland–Altman plots. In our study we could potentially explain the decrease in sum score as most participants were actively undertaking clinical or outpatient multidisciplinary rehabilitation and in medical treatment (92–93%) and the time interval between both questionnaires was rather long, allowing potential functional recovery during the rehabilitation program. However, the large effect size of the Bland Altman analysis at individual level could also indicate that the differences seen in the sum scores of the first versus the second measurement can be attributed to random chance and not be clinically relevant. In our study, the large effect size could imply that the WORQ might not be sensitive enough to monitor change at individual level in clinical practice. At group level, e.g. in research, the WORQ however confirmed good sensitivity to change in this study. Previous studies suggest that the WORQ may be used to measure reliable change in work-related functioning and found Minimal Detectable Change values ranging from 4.3% to 8.95% for the WORQ-FULL and 8.5% for the WORQ-BRIEF [14, 16, 17], representing the change that can be interpreted as real change. These studies calculated the Standard Error of Measurement using Cronbach’s α or person separation index as reliability coefficient. They therefore represent cross-sectional data which are less reliable than our agreement data representing the difference between both measurements.

When analyzing criterion validity and comparing the WORQ-FULL with the WORQ-BRIEF, good correlations were seen (ICC 0.84). The Bland–Altman graph showed that the average item WORQ-score in the WORQ-BRIEF was higher than in the WORQ-FULL. This could be explained by the WORQ-BRIEF having a high proportion of questions on physical complaints and activities resulting in a higher mean score for participants with physical disabilities. Nevertheless, calculating the proportion of dexterity and mobility related items (assumed to be more relevant in persons with physical disabilities) of the WORQ-FULL and -BRIEF (35% respectively 38%), only a slightly higher % is seen in the WORQ-BRIEF [16, 17]. The large limits of agreement and the potential bias seen in the Bland Altman plot emphasize that the full and brief version of the WORQ are better not used interchangeably, but are to be used as separate measurement instruments e.g. when the full version is filled out at intake, also the full version should be used for monitoring progress.

The strong negative correlation of the WORQ-BRIEF with the EuroQoL 5D-5L is interpreted as support for the convergent validity. The correlation of the WORQ-FULL fell just below the cut off level used. The correlation with the WAS was far below the pre-defined cut off value. The correlations of the WORQ have shown varying results in other studies: weak to strong correlations were reported with self-reported general functioning (r = 0.66), HADS anxiety (r = 0.56 and 0.55), HADS depression (r = 0.57 and 0.49), and self-evaluated general health (r = 0.48) [14, 16], the Becks Depression Inventory II (r = 0.51), EuroQol-VAS current health (r = -0.42 and -0.49), the Medical Outcomes Study Short Form-36 (r = -0.35) [10], the work subscales of the Michigan Hand Outcomes Questionnaires (r = -0.25), the work subscales of Disabilities of Arm, Hand and Shoulder (r = 0.28), the WHODAS (r = 0.81) and the WHOQoL (r = -0.47) [16]. Low correlation with the WAS can be explained, because the WAS measures estimated work ability compared with life time best, whereas the WORQ is a presentation of present functions without a comparison. In hindsight, our predefined expectation of a correlation with the WAS may have been incorrect. The high correlation with the EuroQoL 5D-5L in combination with the higher mean score of the WORQ-BRIEF, however, might indicate that the WORQ-BRIEF is a good selection of important items to measure vocational functioning in persons with physical disabilities and therefore a good instrument for use in rehabilitation settings in this population. A loss of information, however, might be expected when using the more practical WORQ-BRIEF only (e.g. in tetraplegia as items on hand function are not included in the WORQ-BRIEF). A previous study mentioned the WORQ-BRIEF can be used as screening instrument on work-related problems, but also mentioned that it is debatable if it is more suitable in research than WORQ [17].

In our study we had a relatively large time interval (max 27 days) possibly resulting in a population with more medical changes over time. However, analysis on both groups with and without medical changes revealed minor differences. Furthermore, our sample size was relatively small and we had a limited number of participants per diagnostic subgroup not allowing analysis per subgroup with sufficient statistical power. In addition, participants completed the WORQ-FULL only and the results from the WORQ-BRIEF were derived from this original instrument. Hence, the actual answers on the shorter version (WORQ-BRIEF) might have been slightly different from the answers on the original version since the answers might be influenced by the presence of other questions.

Future research into the convergent validity should reveal more clarity on the relation of the WORQ with other (work-related) constructs specifically for persons with physical disabilities in a multidisciplinary setting. Also in future studies, a revision of item 34 (driving) could be considered, because many persons in our study (18–20%) have indicated this item as not applicable and it can be interpreted ambiguously. The relevance of this is confirmed by a study from the authors of the WORQ, published whilst our study was conducted, that also opted for the ‘’not applicable’’ answer option on this question [14].

At the moment to our knowledge the WORQ is not used (yet) for clinical practice or in social security settings in the Netherlands. It is still in an experimental phase, hence the importance of this study. Based on the results of our study we would consider using the WORQ-BRIEF in clinical settings as a screening instrument during VR in combination with more diagnose specific instruments investigating work related functioning. We would prefer the brief version above the full version based on the results of the convergent validity study, the very high internal consistency score of the WORQ-FULL (α ≥ 94) indicating potential redundancy of items and the practicality of the WORQ-BRIEF.

In conclusion, we found that the WORQ-NL showed good internal consistency (Cronbach's alpha ≥ 0.85) and test–retest reliability (ICC ≥ 0.85). Agreement demonstrated large score differences are needed to indicate change beyond random chance for all T1 versus T2 measurements at individual level, however at group level small score differences are sufficient. Criterion validity of the WORQ-FULL versus the WORQ-BRIEF was supported (ICC = 0.84) however agreement demonstrated that moderate to large score differences are needed to exceed the limits of agreement indicating change beyond random chance at individual level, however small score differences are sufficient at group level. This indicates the WORQ-FULL and WORQ-BRIEF are better not to be used interchangeably, but are to be used as separate measurement instruments e.g. when the full version is filled out at intake, also the full version should be used for monitoring progress. Convergent validity was supported only for the WORQ-BRIEF with the EuroQoL 5D-5L (r = -0.65).

## Data Availability

The datasets analysed during the current study are available from the corresponding author on reasonable request.
